# Permanent Cure of Hernia

**Published:** 1840-06

**Authors:** 


### PERMANENT CURE OF HERNIA FROM FRACTURE OF THE UPPER EXTREMITY OF THE THIGH BONE.

A distinguished member of the Kentucky bar, when 47 years of age, became affected with scrotal hernia of the right side, for which he was under the necessity of wearing a truss. Judging from the size of the tumor, when the viscera were allowed to descend, the aperture was large. When 57 years old he was overturned in a stage coach, and the upper end of his right thigh bone was fractured. The diagnosis of his medical attendants, was "fracture of the *neck* of the bone," but whether of that part or not, is of no moment, in reference to its effect on the hernia. A great deal of inflammation followed the injury, and when he had so far recovered as to walk about, he found that the hernia did not re-appear. He did not resume his truss, and ten years have since elapsed, without any return of the rupture. In short, the inflammation consequent upon the injury, led to a closure of the hernial aperture. D.

